# Plasma proteomics-based biomarkers for predicting response to mesenchymal stem cell therapy in severe COVID-19

**DOI:** 10.1186/s13287-023-03573-4

**Published:** 2023-12-10

**Authors:** Tian-Tian Li, Wei-Qi Yao, Hai-Bo Dong, Ze-Rui Wang, Zi-Ying Zhang, Meng-Qi Yuan, Lei Shi, Fu-Sheng Wang

**Affiliations:** 1 Senior Department of Infectious Diseases, The Fifth Medical Centre of PLA General Hospital, National Clinical Research Center for Infectious Diseases, No.100 Western 4th Ring Road, Beijing, 100039 People’s Republic of China; 2 Department of Biology and Medicine, Hubei University of Technology, Wuhan, 430030 Hubei People’s Republic of China; 3 Wuhan Optics Valley Zhongyuan Pharmaceutical Co., Ltd., Wuhan, 430030 Hubei People’s Republic of China; 4 Wuhan Optics Valley Vcanbio Cell & Gene Technology Co., Ltd., Wuhan, 430030 Hubei People’s Republic of China; 5 Department of Gastroenterology, First Medical Center of Chinese, PLA General Hospital, Beijing, 100853 People’s Republic of China; 6 Chinese PLA Medical School, Beijing, 100853 People’s Republic of China

**Keywords:** COVID-19, Mesenchymal stem cells, Proteomics, Predictive model

## Abstract

**Background:**

The objective of this study was to identify potential biomarkers for predicting response to MSC therapy by pre-MSC treatment plasma proteomic profile in severe COVID-19 in order to optimize treatment choice.

**Methods:**

A total of 58 patients selected from our previous RCT cohort were enrolled in this study. MSC responders (*n* = 35) were defined as whose resolution of lung consolidation ≥ 51.99% (the median value for resolution of lung consolidation) from pre-MSC to 28 days post-MSC treatment, while non-responders (*n* = 23) were defined as whose resolution of lung consolidation < 51.99%. Plasma before MSC treatment was detected using data-independent acquisition (DIA) proteomics. Multivariate logistic regression analysis was used to identify pre-MSC treatment plasma proteomic biomarkers that might distinguish between responders and non-responders to MSC therapy.

**Results:**

In total, 1101 proteins were identified in plasma. Compared with the non-responders, the responders had three upregulated proteins (CSPG2, CTRB1, and OSCAR) and 10 downregulated proteins (ANXA1, AGRG6, CAPG, DDX55, KV133, LEG10, OXSR1, PICAL, PTGDS, and S100A8) in plasma before MSC treatment. Using logistic regression model, lower levels of DDX55, AGRG6, PICAL, and ANXA1 and higher levels of CTRB1 pre-MSC treatment were predictors of responders to MSC therapy, with AUC of the ROC at 0.910 (95% CI 0.818–1.000) in the training set. In the validation set, AUC of the ROC was 0.767 (95% CI 0.459–1.000).

**Conclusions:**

The responsiveness to MSC therapy appears to depend on baseline level of DDX55, AGRG6, PICAL, CTRB1, and ANXA1. Clinicians should take these factors into consideration when making decision to initiate MSC therapy in patients with severe COVID-19.

**Supplementary Information:**

The online version contains supplementary material available at 10.1186/s13287-023-03573-4.

## Background

Severe acute respiratory coronavirus type 2 (SARS-CoV-2) has caused the ongoing coronavirus disease 2019 (COVID-19) pandemic and has become a prominent public health event worldwide. As of March 2023, the number of infections had reached approximately 762 million, with over 6.8 million deaths [1]. Among them, patients with severe or critical COVID-19 often have lung injury with poor prognosis. Characteristics of lung injury on chest CT appear as ground-glass opacity, consolidation, crazy-paving pattern, etc. Consolidation on chest CT refers to the replacement of alveolar air with pathological fluids, cells, or tissues, as indicated by an increase in pulmonary parenchymal density that obscures the borders of underlying arteries and airway walls [2]. For patients with COVID-19, consolidation was also thought to be an indicator of disease progression [3]. The prognosis of CT consolidation varies by individual, and some patients will develop post-acute fibrosis [4]. The sequelae, such as lung injury, after recovery from acute COVID-19 are severe threats for survivors [5]. New therapeutic strategies are vital for the treatment of COVID-19, especially for severe or critical patients.

Mesenchymal stem cells (MSC) have been shown to play therapeutic roles in acute lung injury, ARDS, and lung fibrosis, owing to their anti-inflammatory and immunomodulatory properties [6, 7]. As of March 2023, more than 90 clinical studies of MSC for COVID-19 have been registered at clinicaltrials.gov [8, 9]. Although preliminary clinical trial data have demonstrated good safety and encouraging efficacy of MSC for COVID-19, some patients still fail to respond to MSC therapy. Clinical studies must, therefore, establish strategies to identify biological characteristics of these potential responders to MSC therapy in order to optimize treatment choice.

Plasma has a dynamic range of protein abundance that exceeds ten orders of magnitude, making it an excellent source of biological information [10]. Mass spectrometry (MS) is a powerful tool for analyzing intact protein from plasma. Systematic omics studies, such as proteomics, provide a comprehensive understanding of biological processes in patients and facilitate in the discovery of biomarkers [11–13].

In March 2020, we performed a multicenter, randomized, double-blind phase II study of MSC therapy in patients with severe COVID-19 (ClinicalTrials.gov: NCT04288102). Findings showed that MSC was a potentially effective therapeutic approach for patients with severe COVID-19 [14, 15], but not all patients responded well. To further understand the proteomic biomarkers of response to MSC therapy in patients with severe COVID-19, we characterized the proteomic profile of 58 patients pre-MSC treatment plasma samples (35 responders versus 23 non-responders) by using proteomics assays. The present study aimed to identify potential biomarkers for predicting response to MSC therapy by pre-MSC treatment plasma proteomic profile in severe COVID-19.

## Methods

### Study design

This prospective longitudinal cohort study aimed to identify potential proteomic biomarkers pre-MSC treatment for predicting response to MSC therapy in severe COVID-19. Patients and data were from MSC group of our previous randomized trial, which enrolled between March 6, 2020, and March 20, 2020, at two hospitals in Wuhan, Hubei, China (NCT04288102). First, we identified and compared the differentially expressed proteins between the responders and non-responders. The proteomic data from this cohort were randomly divided into training and validation sets with a 4:1 ratio to build a prediction model and then evaluate with receiver operating characteristic (ROC) methods.

### Participants and groups

As previously described [14, 15], 66 patients in the MSC treatment group and 35 patients in the placebo group were enrolled in our previous multicenter, randomized, double-blind phase II trial of MSC therapy in patients with severe COVID-19. Inclusion criteria were hospitalized patients with severe COVID-19 confirmed by real-time reverse transcription PCR assay, either man or woman, aged 18–75 years old. Patients had pneumonia combined with lung damage confirmed by chest CT. Exclusion criteria were shock, organ failures, invasive ventilation, malignant tumor, pregnancy, lactation, or co-infection of other pathogens. Human umbilical cord MSCs (with each dose of 4.0 × 10^7^ cells) or placebo were infused for severe COVID-19 patients intravenously three times at 3-day intervals. The endpoint was the percentage decline rates of consolidation volume to whole lung volume on high-resolution chest computed tomography (HRCT) from pre-MSC treatment to 28 days post-MSC treatment, which measured by centralized imaging interpretation based on imaging software (LIAIS). LIAIS-assisted lung volumetry and densitometry procedure was as described in our previous report [14]. Briefly, consolidation volume was segmented automatically after importing raw CT images to the software. Subsequently, segmentation was corrected by three independent reviewers on the software platform. The percentage decline rates of consolidation volume were defined as (consolidation proportion of the whole lung volume at day 28-consolidation proportion of the whole lung volume pre-MSC treatment)/(consolidation proportion of the whole lung volume pre-MSC treatment).

**Fig. 1:**
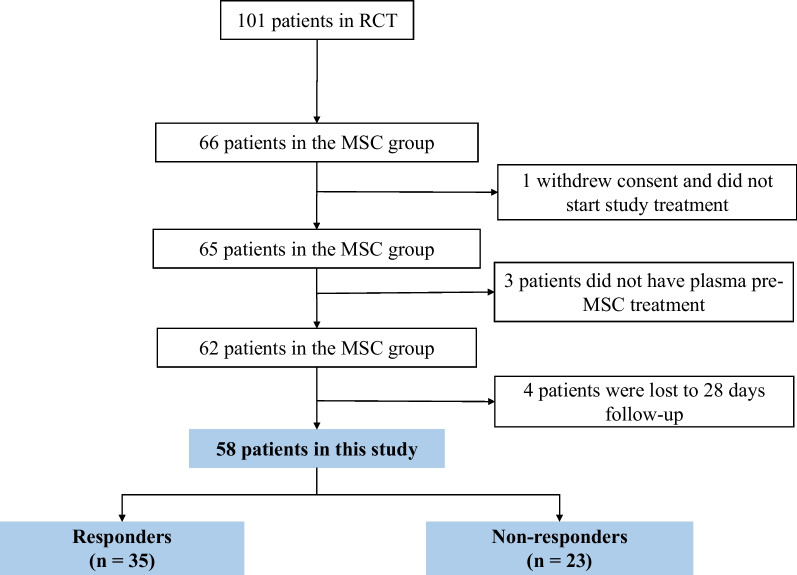
Participants in this study

In this study, patients were selected from the 66 patients of the MSC group. Of which, one patient withdrew consent, three patients had no plasma pre-MSC treatment, and four patients lost to follow-up at day 28. Eventually, a total of 58 patients were included in this study (Fig. 1). The previous results showed that MSC significantly promoted the resolution of lung consolidation at day 28, but not for all patients. The median value for resolution of lung consolidation was 51.99%. Therefore, in this study, we used the median value of resolution of lung consolidation for all enrolled patients as the cutoff value and classified the patients into responders (*n* = 35) and non-responders (*n* = 23). MSC responders were defined as whose resolution of lung consolidation ≥ 51.99%, while non-responders were defined as whose resolution of lung consolidation < 51.99%.

Ethical approval was obtained from the Ethics Committee of the Fifth Medical Center, Chinese PLA General Hospital (2020-013-D). Written informed consent was obtained from all enrolled patients. Demographic information, clinical characteristics, laboratory examinations, and CT results were obtained using the electronic data capture system.

### Samples

On the morning prior to MSC treatment, peripheral blood samples were collected on the median cubital vein by venipuncture in 10-mL vacutainer tubes containing EDTA, subsequently mixed well by using gentle inversion. Plasma was separated by centrifugation at 2500*g* for 10 min at 4 °C and stored at − 80 °C for further proteomic analysis.

### Liquid chromatography with tandem mass spectrometry (LC–MS/MS)

Proteomics assays were performed using the data-independent acquisition (DIA) method. Total proteins in plasma were extracted, denatured, and digested into peptides using trypsin. A Orbitrap Exploris 480 mass spectrometer was used in conjunction with an Easy-nLC 1200 system to collect LC–MS/MS data. Peptides were separated on a C18 analytical column (75 μm × 25 cm, C18, 1.9 μm, 100 A), and the gradient was established using mobile phase A (0.1% formic acid) and mobile phase B (80% ACN, 0.1% formic acid) at a flow rate of 300 nL/min. Each scan cycle for DIA mode analysis includes one full-scan mass spectrum (*R* = 60 K, AGC = 3e6, Max IT = 30 ms, scan range = 350–1250 m/z) followed by 40 variable MS/MS events (*R* = 30 K, AGC = 1000%, Max IT = 50 ms), with FAIMS CV voltage of − 45 and HCD collision energy of 30.

The library-free MS data analysis was processed using DIA-NN software (version 1.8). Deep learning methods were used to predict libraries using the SwissProt human protein sequence database. A spectrum library constructed from DIA data using the MBR function was used for data reanalysis, with a final precursor and protein FDR of 1%. The DIA-NN output files providing quantification information for the protein groups were subsequently utilized. Proteins detected in more than 50% of the samples in at least one category were first grouped and filtered. Missing values were imputed using values from a normal distribution around the detection limit of the mass spectrometer. The mean and standard deviation of the real intensity distribution were calculated, and a new distribution with a 1.8-standard deviation downshift and 0.25-standard deviation width was generated. These values were used to impute the entire matrix, allowing statistical analysis.

### Differentially expressed proteins and functional annotation

Differentially expressed proteins (DEPs) were defined as those with a fold change (FC) > 1.5 or FC < 1/1.5 and *p* < 0.05 between the responders and non-responders. The “clusterProfiler” package was adopted to execute enrichment analysis in DEPs, including the Kyoto Encyclopedia of Genes and Genomes (KEGG) assessment and gene ontology (GO) assessment (biological process (BP), molecular function (MF), and cellular component (CC)).

### Least absolute shrinkage and selection operator regression analysis

To optimize the latent collinearity and avoid over-fitting of variables, the least absolute shrinkage and selection operator (LASSO) regression analysis was subsequently used to further screen the most significant proteins using the R software package “glmnet.”

### Multivariate logistic regression analysis

**Fig. 2:**
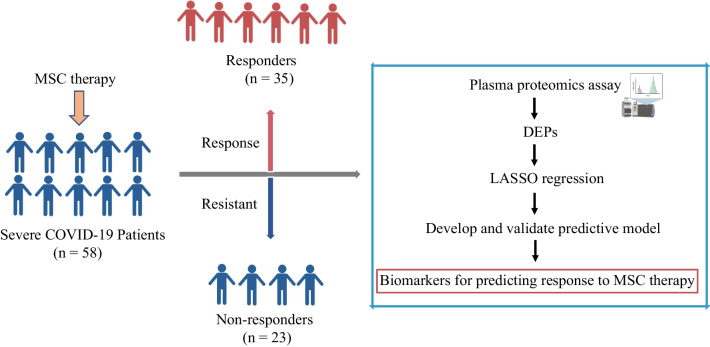
Study flowchart. Abbreviations: LASSO, least absolute shrinkage and selection operator

LASSO-selected proteins were subsequently included in the multivariate logistic regression analysis. Multivariate logistic regression analysis was used to identify pre-MSC treatment plasma proteomic biomarkers that might distinguish between responders and non-responders to MSC therapy at 28 days. Fifty-eight patients were divided into a training set (*n* = 47) and a validation set (*n* = 11) in a 4:1 ratio using the stratified random sampling method with R caret package [16, 17]. Subsequently, a multiple biomarker panel was developed using the training set data. Fivefold cross-validation was used in the training set. Finally, the performance of the multiple biomarker panel was verified using the validation set (Fig. 2).

### Statistical analysis

Continuous variables were presented as mean with standard deviation and analyzed using an unpaired Student’s *t*-test for normally distributed variables or median with interquartile range (IQR) and analyzed using a Mann–Whitney *U*-test for skewed data. Categorical variables were expressed as absolute numbers and percentages and were analyzed using the Chi-square or Fisher’s exact test. Other statistical analyses included the ROC curve, confusion matrix, nomogram, and calibration plot. For all tests, a two-tailed *p* < 0.05 was considered statistically significant. Data were analyzed using R (version 4.1.2) and Python (version 3.9.0).

## Results

### Baseline clinical characteristics and 28 days outcomes in the MSC group

Regarding baseline clinical characteristics, 58 COVID-19 patients in the present study had a median age of 60.10 years, including 33 (56.90%) men. The median time from symptom onset to MSC treatment was 46 days (IQR, 39.50-51.75). Fever was the most common symptom, followed by cough. The top four comorbidities were hypertension, diabetes, chronic bronchitis, and chronic obstructive pulmonary disease, with 26 patients being free of additional conditions. Commonly used drugs included antivirals, antibiotics, and corticosteroids. The baseline clinical characteristics between the non-responders and responders were similar in terms of sex, age, and comorbidities (Table 1). Regarding 28 days outcomes, the median value for resolution of lung consolidation in the non-responders was − 24.20% (23 patients) and − 71.93% in the responders (35 patients). At day 28, none of the patients died, and all nucleic acid tests were negative.

**Table 1:** Baseline demographic and clinical characteristics in the MSC group

	Training set (*n* = 47)			Validation set (*n* = 11)		
	Non-responders	Responders	*p*	Non-responders	Responders	*p*
	(*n* = 17)	(*n* = 30)		(*n* = 6)	(*n* = 5)	
Sex, *n* (%)			0.766			0.455
Male	8 (47.06)	16 (53.33)		4 (66.67)	5 (100.00)	
Female	9 (52.94)	14 (46.67)		2 (33.33)	0 (0.00)	
Age (years)	59.20 (8.74)	58.75 (10.06)	0.795	64.17 (9.97)	54.75 (8.73)	0.188
BMI (kg/m^2^)	24.10 [21.90, 26.50]	26.00 [23.18, 27.68]	0.293	25.40 [24.08, 27.78]	26.15 [25.05, 26.88]	0.855
Comorbidities, *n* (%)			1.000			1.000
No	8 (47.06)	14 (46.67)		2 (33.33)	2 (40.00)	
Yes	9 (52.94)	16 (53.33)		4 (66.67)	3 (60.00)	
White blood cell count (× 10^9^/L)	5.90 [5.00, 7.30]	5.60 [4.78, 7.10]	0.756	5.85 [5.43, 6.68]	5.50 [5.23, 5.93]	0.646
Neutrophil cell count (× 10^9^/L)	3.49 [2.85, 4.97]	3.57 [2.83, 4.38]	0.842	3.70 [3.07, 4.50]	3.38 [3.21, 3.64]	0.465
Lymphocyte cell count (× 10^9^/L)	1.65 (0.47)	1.43 (0.51)	0.124	1.54 (0.34)	1.44 (0.38)	0.933
Platelet count (× 10^9^/L)	250.20 (80.01)	230.00 (79.28)	0.478	196.33 (16.35)	214.25 (46.62)	0.277
IL-6 (pg/mL)	9.33 [8.08, 17.60]	7.32 [4.31, 11.43]	0.128	6.59 [5.79, 9.04]	6.75 [6.20, 7.68]	0.854
Time from symptom onset to MSC treatment (days)	47.00 [43.00, 53.00]	45.00 [34.25, 47.00]	0.173	55.50 [34.00, 57.25]	42.50 [29.50, 52.50]	0.233
Smoking, *n* (%)						
No	15 (88.24)	27 (90.00)	1.000	6 (100.00)	5 (100.00)	–
Yes	2 (11.76)	3 (10.00)		0 (0.00)	0 (0.00)	
Drinking, *n* (%)						
No	16 (94.12)	29 (96.67)	1.000	6 (100.00)	5 (100.00)	–
Yes	1 (5.88)	1 (3.33)		0 (0.00)	0 (0.00)	
Oxygen therapy, *n* (%)						
No	3 (17.65)	3 (10.00)	0.653	1 (16.67)	1 (20.00)	1.000
Yes	14 (82.35)	27 (90.00)		5 (83.33)	4 (80.00)	
RBC count (× 10^9^/L)	4.01 [3.80, 4.43]	3.93 [3.45, 4.28]	0.173	4.10 [3.67, 4.45]	4.49 [4.22, 4.67]	0.465
Albumin (g/L)	37.91 (2.59)	36.46 (3.29)	0.054	36.23 (3.16)	36.80 (2.12)	0.763
Globulin (g/L)	29.19 (5.29)	30.02 (4.57)	0.443	29.27 (5.04)	29.63 (4.47)	0.911
The percentage decline rates of consolidation volume to whole lung volume on CT from pre-MSC treatment to 28 days	− 21.72 [− 41.21, − 10.13]	− 71.61 [− 87.51, − 62.34]		− 27.91 [− 37.96, 34.11]	− 81.12 [− 96.37, − 75.40]	

Values are n (%), mean (SD), or median [IQR] for skewed data

*BMI* body mass index, *IL-6* interleukin-6, and *RBC* red blood cell count

### Differentially expressed proteins in plasma before MSC treatment between responders and non-responders

**Fig. 3:**
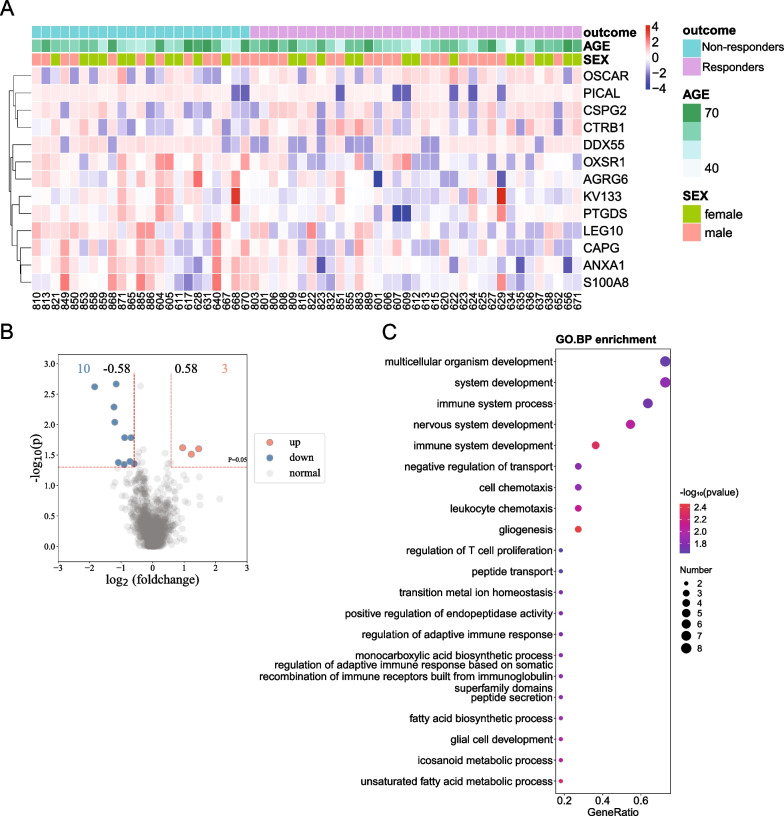
Thirteen differentially expressed proteins (DEPs) and their functional enrichment analysis between the responders and non-responders pre-MSC treatment. **A** Heatmap showing 13 differentially expressed proteins between the responders and non-responders. **B** The red dots represent the upregulated DEPs based on *p* < 0.05 and fold change (FC) > 1.5, and the blue dots represent downregulated DEPs based on *p* < 0.05 and FC < 1/1.5. The gray spots represent proteins with no significant difference. **C** GO-based enrichment analysis of DEPs in terms of biological processes (hypergeometric test; *p* < 0.05). GO terms were sorted according to *p*

**Fig. 4:**
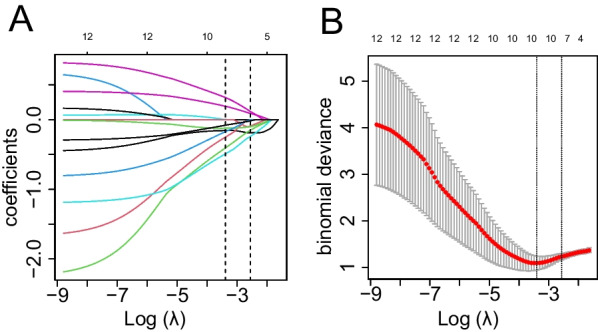
Selection of 10 proteins using LASSO binary logistic regression model between the responders and non-responders from 13 DEPs. **A** LASSO coefficient profiles of the 13 proteins. Vertical line was drawn at the value selected using fivefold cross-validation, where optimal λ resulted in 10 nonzero coefficients.** B** Tuning parameter (*λ*) selection in the LASSO model used fivefold cross-validation via minimum partial likelihood deviance. Abbreviations: LASSO, least absolute shrinkage and selection operator

In order to obtain a comprehensive understanding of plasma protein levels in enrolled patients, proteomic analysis was performed. A total of 1101 proteins were identified by proteomics analysis of the plasma in the patients. As shown in Additional file 1: Fig. S1, the distribution and quality control of the total peptides and proteins detected indicated that the proteomics data were of good quality and reproducibility. Subsequently, a comparison of plasma protein levels was performed between the responders and non-responders. Proteome screening identified 13 differentially expressed proteins in plasma with significant FC and *p* values between the responders and non-responders (Table 2 and Fig. 3A). As shown in Fig. 3B, the responders had three upregulated proteins (CSPG2, CTRB1, and OSCAR) and 10 downregulated proteins (ANXA1, AGRG6, CAPG, DDX55, KV133, LEG10, OXSR1, PICAL, PTGDS, and S100A8) compared with that in the non-responders. The subsequent GO BP analysis of these 13 proteins indicated that their functions were mainly enriched in multicellular organism development, system development, immune system process, and nervous system development sorted according to the gene ratio and *p* values (Fig. 3C). GO CC analysis revealed significant enrichment in the extrinsic components of the membrane and plasma membrane. GO MF analysis revealed significant enrichment in the anion, lipid, and phospholipid binding. The KEGG enrichment was primarily related to arachidonic acid metabolism. These 13 proteins were then subjected to LASSO regression analysis, among which 10 were chosen (Fig. 4), with the names and coefficients of the proteins shown in Additional file 2: Table S1.

**Table 2:** Up-/down-regulated differentially expressed proteins (DEPs) between the responders and non-responders pre-MSC treatment

Protein names	UniprotKB	Gene names	Mean (responders)	Mean (non-responders)	*p*	Up-/down-regulated
CSPG2	P13611	VCAN	15.481	14.252	0.031	Up
**CTRB1**	**P17538**	**CTRB1**	**15.494**	**14.540**	**0.024**	**Up**
OSCAR	Q8IYS5	OSCAR	14.718	13.254	0.025	Up
**ANXA1**	**P04083**	**ANXA1**	**15.973**	**17.200**	**0.005**	**Down**
**AGRG6**	**Q86SQ4**	**ADGRG6**	**16.955**	**17.639**	**0.016**	**Down**
CAPG	P40121	CAPG	13.617	14.336	0.040	Down
**DDX55**	**Q8NHQ9**	**DDX55**	**17.657**	**19.497**	**0.002**	**Down**
KV133	P01593	IGKV1-33	20.221	21.109	0.016	Down
LEG10	Q05315	CLC	15.315	16.517	0.009	Down
OXSR1	O95747	OXSR1	12.997	14.154	0.002	Down
**PICAL**	**Q13492**	**PICALM**	**20.039**	**21.127**	**0.042**	**Down**
PTGDS	P41222	PTGDS	17.964	18.867	0.045	Down
S100A8	P05109	S100A8	21.790	22.381	0.044	Down

Differentially expressed proteins (DEPs) are defined as those proteins with a fold change (FC) > 1.5 or FC < 1/1.5 and *p* < 0.05 between the responders and non-responders. Upregulated proteins in the responders compared with that in the non-responders, fold change (FC) > 1.5 and *p* < 0.05. Downregulated proteins in the responders compared with that in the non-responders, FC < 1/1.5 and *p* < 0.05. Words highlighted in bold indicate that the five proteins identified in the final logistic regression model

### Potential proteomic biomarkers for predicting response to MSC therapy

**Fig. 5:**
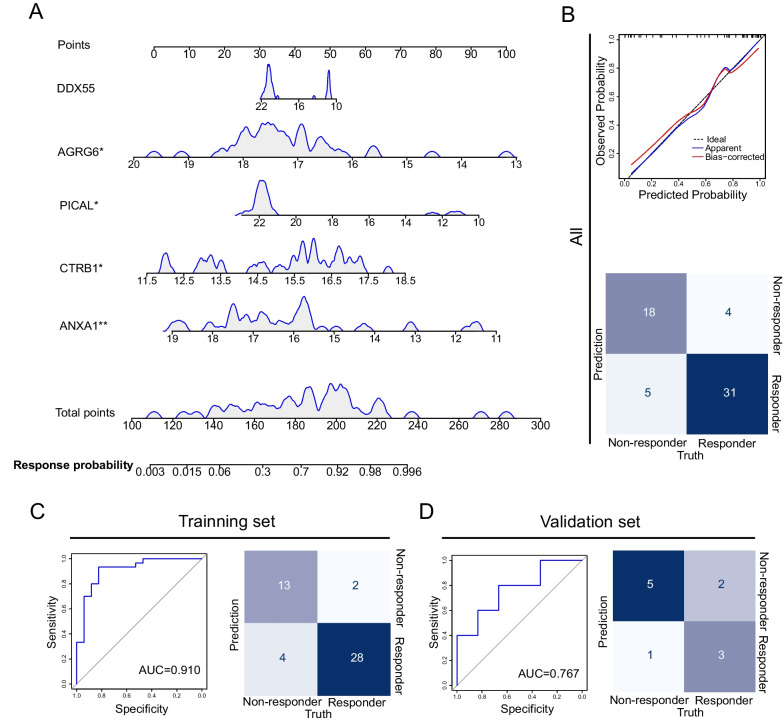
Related biomarkers of lung pathological recovery in severe COVID-19 patients in the MSC group.**A** Nomogram of five protein combinations to predict the percentage decline rate of consolidation ≤  − 51.99% after MSC therapy (28-day good outcome). To use the nomogram, the protein expression level of an individual value is located on each variable axis, and a line is drawn upward to determine the number of points received for each variable protein expression level. The sum of these numbers is located on the total points axis. A line is drawn downward to the 28-day good outcome probability axes to determine the probability of the percentage decline rate of consolidation ≤  − 51.99% after MSC therapy. **B** Calibration plot and confusion matrix to assess the accuracy of the model in 58 patients. **C** The receiver operating characteristic (ROC) curve and confusion matrix to distinguish responders from non-responders outcomes in the training set. The ROC curves were created by plotting the sensitivity (i.e., true-positive rate) against 1 − specificity (i.e., false-positive rate). The line in each plot represents the area under the curve (AUC). **D** ROC curve and confusion matrix to distinguish responders from non-responders outcomes after MSC therapy in the validation set-1

To construct a concise prediction biomarker panel, no more than five random combinations of the 10 proteins screened by LASSO regression were chosen as candidates for predictive model. Based on the training set, an ideal panel was defined as a combination of DDX55, AGRG6, PICAL, CTRB1, and ANXA1. Finally, multivariate logistic regression identified AGRG6, PICAL, CTRB1, and ANXA1 as independent factors that predicted response to MSC therapy in patients with severe COVID-19. Low levels of AGRG6, PICAL, and ANXA1, as well as elevated levels of CTRB1, were associated with responsiveness to MSC therapy (Table 3). Figure 5A shows the predictive nomogram with weights and points. The calibration plot revealed the best agreement between the nomogram predictions and actual observations (Fig. 5B). The area under the curve (AUC) of the ROC for the training set of responders vs. non-responders was 0.910 (95% CI 0.818–1.000), for the validation set 0.767 (95% CI 0.459–1.000) (Fig. 5C and D), indicating that this model was accurate to predict responsiveness.

**Table 3:** Final model of the multivariate logistic regression analysis in the training set

Protein names	B	Wald	OR (95% CI)	*p*	Estimate	Std. error	*Z* value
DDX55	− 0.160	1.761	0.852 (0.674–1.078)	0.184	− 0.160	0.120	− 1.327
AGRG6	− 1.396	4.439	0.248 (0.068–0.908)	0.035	− 1.396	0.663	− 2.107
PICAL	− 0.468	4.674	0.626 (0.410–0.956)	0.031	− 0.468	0.216	− 2.162
CTRB1	0.944	4.687	2.570 (1.094–6.041)	0.030	0.944	0.436	2.165
ANXA1	− 1.036	7.667	0.355 (0.170–0.739)	0.006	− 1.036	0.374	− 2.769

## Discussion

MSC transfusion has been found to improve the restoration of lung injury in severe cases with COVID-19; however, only some of the treated COVID-19 patients responded well to MSC treatment [14]. In this context, predicting the response to MSC and selecting potential responders before MSC therapy is highly desirable. The mechanism of MSC treatment may involve multiple targets comprising anti-inflammatory, immunomodulatory, and regeneration. MS examines the proteome as a whole. Therefore, we believe that a systematic proteomic approach is necessary to identify multiple biomarkers. Our results showed that low levels of DDX55, ANXA1, PICAL, and AGRG6 before treatment, as well as elevated levels of CTRB1 pre-MSC treatment, were biomarkers for predicting response to MSC therapy in severe COVID-19 patients. Therefore, the assessment of such proteins before MSC may be a new promising approach to identify potential respondents and give them a better chance of attaining full recovery after MSC therapy.

In this study, we tried to develop a predictive model using five selected proteomic markers and found that this model could discriminate responders from non-responders. Several previous prognostic models for COVID-19 have mostly been used to predict multiple outcomes after stand care, including disease progression, acute respiratory distress syndrome, admission to ICU, death, duration of mechanical ventilation, complications of cardiac injury, and thrombosis [18]. However, none of the predictive models used to guide patients will benefit from MSC treatment in COVID-19 before this study. Since this reason, we cannot know whether the sensitivity and specificity of this model were superior to others. Notably, our results indicated that compared to just one marker (DDX55, ANXA1, PICAL, AGRG6, or CTRB1), the discrimination potential of the five markers panel was found to be superior.

The associated mechanisms responsible for the decreased levels of DDX55, ANXA1, PICAL, and AGRG6 before treatment and the elevated level of CTRB1 pre-MSC treatment with responsiveness to MSC therapy are still unclear. As the above-mentioned proteins play a role in physiological and pathological functions, it is now recognized that the investigation of proteins function is essential to obtain an accurate understanding of disease pathogenesis. ANXA1, also known as lipocortin-1, is a member of a family of proteins that bind to membrane phospholipids, resulting in the inhibition of phospholipase A2 and eicosanoid production [19]. It is an endogenous suppression modulator of inflammation expressed in monocytes and neutrophils [20]. ANXA1 is reportedly involved in SARS-CoV-2 infection, especially in patients with severe disease, through interactions with complement molecules and lipids, mediating a systemic cytokine storm [21, 22]. A case–control study demonstrated that Annexin A1 is a potential prognostic biomarker in the diagnosis of COVID-19 pneumonia and in predicting the need for ICU treatment in patients with COVID-19 [23]. In this study, we found that patients with low ANXA1 levels were more likely to respond to MSC treatment and have a better prognosis. This may be because patients with low ANXA1 levels before treatment have weak interactions with complement molecules and lipids, mediating a low cytokine response in COVID-19. The level of ANXA1 is regulated by exogenous drugs and cells such as ingested glucocorticoids [24–26] and MSC paracrine [27]. PICAL is a cytoplasmic adapter protein that plays a critical role in clathrin-mediated endocytosis and is found in the nasopharynx, bronchial, and lung tissues. The depletion of this receptor has previously been shown to inhibit clathrin-mediated endocytosis [28]. In COVID-19, it has been established that both clathrin-mediated and clathrin-/caveolae-independent endocytosis are an essential mechanism for the internalization of SARS-CoV [29]. AGRG6/Adgrg6, also named GPR126, is a member of the adhesion G-protein-coupled receptor family of proteins involved in cell adhesion and signaling. Although the precise function of this protein has not been elucidated, its expression is highest in the adult lungs [30]. It has been reported that AGRG6 polymorphisms are associated with FEV1/FVC at genome-wide significance [31]. At present, studies on CTRB1 are mainly focused on pancreas-related diseases, and its role in lung diseases needs to be further explored. We believe that the elucidation of the underlying mechanism between these proteins and response to MSC therapy in COVID-19 might also provide potential targets for new therapeutic strategies.

The dynamic changes in the plasma proteins after MSC infusion, including these five proteomic markers, are of great interest. MSC infusion reduced the levels of TGF-β, TNF-α, type I and III collagen, C-reactive protein, and neutrophil extracellular traps, while increased IDO, PGE2, IL-10, IL-4, TGF-α, VEGF, FGF, HGF, and KGF [32, 33]. Further study will be necessary. Particularly, it would be interesting to see whether the dynamic change of these proteins has enhanced the performance of prediction models for responsiveness to MSC treatment.

There were several limitations to this study. First, the sample size was small, and a larger sample size is necessary for further validation. Second, an internal cohort was used for model validation. These findings must be further verified in external validation cohorts before they can be used in clinical settings.

## Conclusions

The responsiveness to MSC therapy appears to depend on baseline level of DDX55, AGRG6, PICAL, CTRB1, and ANXA1. Clinicians should take these factors into consideration when making a decision to initiate MSC therapy in patients with severe COVID-19. In the future, external validation cohorts and large prospective studies are needed to confirm the preliminary findings of this study. It is also necessary to gain a better understanding of the longitudinal dynamics of plasma proteomic markers during MSC intervention.

### Supplementary Information

**Additional file 1**. **Fig. S1**: Distribution and quality control of the total proteins detected.

**Additional file 2**. **Table S1**: Coefficients of 10 proteins screened by LASSO regression.

## Data Availability

The mass spectrometry proteomics data generated during the current study are available in the ProteomeXchange Consortium via the iProX repository with the dataset identifier PXD042106.

## Abbreviations

SARS-CoV-2: Severe acute respiratory coronavirus type 2
COVID-19: Coronavirus disease 2019
ARDS: Acute respiratory distress syndrome
MSC: Mesenchymal stem cells
MS: Mass spectrometry
DIA: Data-independent acquisition
DEPs: Differentially expressed proteins
FC: Fold change
KEGG: Kyoto Encyclopedia of Genes and Genomes
GO: Gene ontology
BP: Biological process
MF: Molecular function
CC: Cellular component
LASSO: Least absolute shrinkage and selection operator
ROC: Receiver operating characteristic curve
CSPG2: Versican core protein
CTRB1: Chymotrypsinogen B1
OSCAR: Osteoclast-associated immunoglobulin-like receptor
ANXA1: Annexin A1
AGRG6: Adhesion G-protein-coupled receptor G6
CAPG: Macrophage capping protein
DDX55: DEAD-box helicase 55
KV133: Immunoglobulin kappa variable 1D-33
LEG10: Galectin-10
OXSR1: Serine/threonine protein kinase OSR1
PICAL: Phosphatidylinositol-binding clathrin assembly protein
PTGDS: Prostaglandin-H2 D-isomerase
S100A8: Protein S100-A8
AUC: Area under the curve
