# Supplementary material for: Plasma proteomics-based biomarkers for predicting response to mesenchymal stem cell therapy in severe COVID-19
Source: Stem Cell Res Ther. 2023 Dec 10;14:350. doi: 10.1186/s13287-023-03573-4 (PMC10712100; doi:10.1186/s13287-023-03573-4)
Supplement: Supplementary file 1 — Additional file 1. Fig. S1: Distribution and quality control of the total proteins detected. [file 13287_2023_3573_MOESM1_ESM.pdf]

Additional file 1

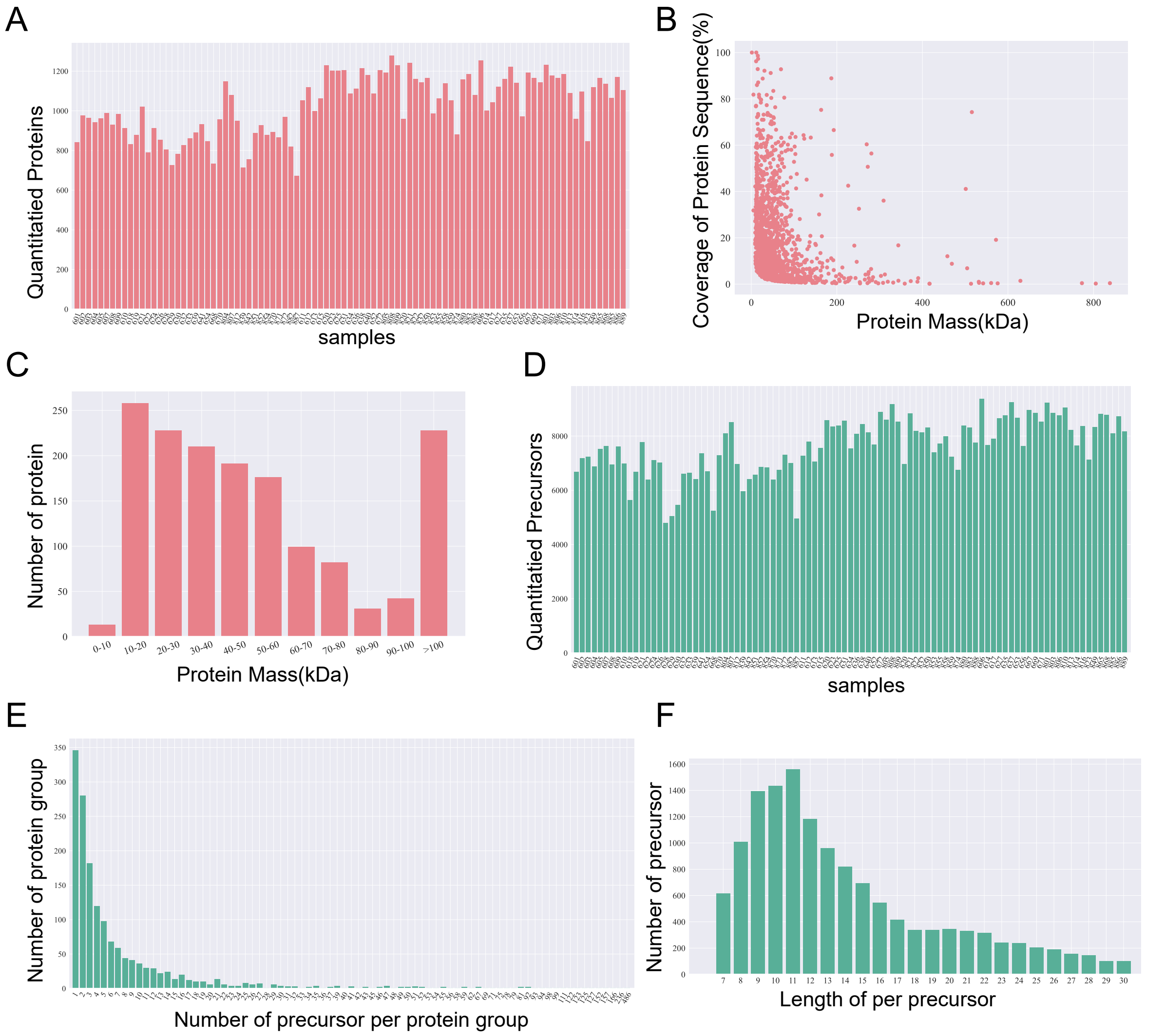

Fig. S1 Distribution and quality control of the total proteins detected. **A** The distribution of numbers of quantified proteins in plasma samples. **B** The distribution of coverage of proteins sequence and mass. **C** The distribution of numbers of proteins in different protein masses. **D** The distribution of numbers of quantified precursors in plasma samples. **E** The distribution of the number of protein groups in the different number of precursors per protein group. **F** The distribution of the number of precursors in different lengths per precursor.
