# Supplementary material for: Plasma proteomics-based biomarkers for predicting response to mesenchymal stem cell therapy in severe COVID-19
Source: Stem Cell Res Ther. 2023 Dec 10;14:350. doi: 10.1186/s13287-023-03573-4 (PMC10712100; doi:10.1186/s13287-023-03573-4)
Supplement: Supplementary file 2 — Additional file 2. Table S1: Coefficients of 10 proteins screened by LASSO regression. [file 13287_2023_3573_MOESM2_ESM.docx]

**Additional file 2**

**Table S1** Coefficients of 10 proteins screened by LASSO regression.

| **Protein names** | **coefficients** |
| --- | --- |
| CSPG2 | 0.002 |
| CTRB1 | 0.319 |
| OSCAR | 0.193 |
| ANXA1 | -0.408 |
| AGRG6 | -0.493 |
| DDX55 | -0.080 |
| KV133 | -0.253 |
| LEG10 | -0.123 |
| OXSR1 | -0.156 |
| PICAL | -0.173 |
